# Lessons from COVID-19 on the role of the state and the market in providing early testing

**DOI:** 10.7189/jogh.10.020330

**Published:** 2020-12

**Authors:** Chantal M Morel, Olof Lindahl, Volkan Özenci

**Affiliations:** 1 GTGL, University of Geneva, Genève, Switzerland; 2 Department of Business Studies, Uppsala University, Uppsala, Sweden; 3 Department of Laboratory Medicine, Karolinska Institute, Stockholm, Sweden

Responses to the current COVID-19 pandemic clearly demonstrate that, without confirmed diagnosis, we are effectively fumbling in the dark – and doing so with major clinical, economic and social consequences. Yet getting the right diagnostics is not instantaneous and requires protocols and sufficient standing capacity in order to be able to test people in large numbers at the outset of an epidemic. This paper examines the roles of government and industry in producing diagnostic tools on demand and explores how we could improve prompt development and availability of these technologies to help stem infectious disease epidemics earlier in the future. Better preparation and coordination will be challenging, very expensive, result in some seemingly unnecessary public spending to hedge against risk, and require that states grant greater independence to an international institution who can counter the harmful effects of national self-prioritization.

## GLOBAL RESPONSES TO COVID-19 HAVE LED TO VERY DIFFERENT LEVELS OF SUCCESS

COVID-19 control interventions in Europe, as in many places across the world, have mainly revolved around case-based self-isolation, social distancing, banning public events, closing schools, and ordering home lock-down. While these measures are estimated to have had some success in lowering the death rate from the disease [1]. they have also been damaging to the global economy and it remains to be seen how fast it can recover. One need only look at the desperate spending attempts to keep national economies afloat to understand the magnitude of the expected impact: the US$2 trillion government stimulus package in the United States (US), or the €1.350 billion (approximately US$1.485 billion) pandemic emergency purchase programme launched by the European Central Bank, or the £105billion (approximately US$135 billion) immediate fiscal impulse in the United Kingdom (UK).

In addition to their enormous economic cost, the social isolation measures taken in Europe and the US are largely deficient, last resort measures. Even where they are strictly enforced, there remains some flow of potentially infected or contaminated individuals, including health workers, transport workers, police, delivery personnel, etc. So, even where people are ostensibly isolated from one another, lock-down as we know it remains porous. And as we do not know who is infected, who is not infected, or who has any immunity to the infection, there is little meaningful data on which we can justify a shift back to normal life. The population and the economy are effectively left waiting for news of a vaccine, which will take many months or perhaps a few years.

Yet, as we know, not every country is in this same situation. Two that have based their quarantine measures on mass diagnosis have fared much better thus far. Indeed Singapore and South Korea have seemingly managed to minimize both the human and economic cost of COVID-19 by determining who was infected in the early phase. The key to this success was the early availability and widespread use of molecular diagnostic testing. Germany has also done relatively well overall due to its focus on early testing (see below).

## THE NEED FOR LABORATORY METHODS TO BE IN PLACE AND ON TIME

Authorities in Wuhan province first identified the new pathogen, SARS-CoV-2, using laboratory-based reverse transcription polymerase chain reaction (RT-PCR) when presented with a patient with pneumonia of unknown aetiology [2]. Molecular testing such as with RT-PCR is indeed the norm for identifying emerging infectious diseases [3]. It tells us if there is a pathogen and allows us to match it against known pathogens to understand its origin. In this case, the samples proved positive for pan-Betacoronavirus and whole genome sequencing followed by bioinformatic analyses indicated that the virus had typical features of a coronavirus belonging to the 2B lineage and was closest to the bat SARS-like coronavirus strain BatCov RaTG13 [4]. PCR technology, which is very commonly used in microbiology laboratories, can detect even a tiny amount of viral ribonucleic acid and establish the presence of infection in both symptomatic and asymptomatic patients.

Not long after publication of the full genome sequence of SARS-CoV-2 by the Chinese, microbiologists around the world started designing, testing, and publishing protocols for detecting the new virus. The World Health Organization (WHO) published these protocols on their website such that other laboratories could test efficiently for the virus. As the virus made its way west, most countries tested symptomatic patients presenting to hospital, also using laboratory-based PCR methods, customized using different primers that target different sections of the SARS-CoV-2 genome [5].

Such hospital analyses are of course useful for understanding the virus’ transmission pattern across the globe. They provide some measure for how much a country is affected compared to others (those with similar testing policies) and allow us to gauge the rate of growth in severe cases and fatalities. On an individual level these analyses also help inform patient management. However, the number of cases detected by these tests is believed to be orders of magnitude lower than the number of true infections due to the number of mild and asymptomatic cases that are not formally identified and the limited overall testing capacity [6]. Crucially, hospital-only use of these molecular tests does not give us information necessary to prevent the propagation of the virus or its impact on the wider population. It also does not provide any meaningful estimation of the death rate beyond the proportion of those presenting with symptoms at hospitals who eventually die. This makes it very hard to know how to plan – for example how many ventilators will be needed.

In contrast, Singapore and South Korea used the same molecular technology within a wide-spread screening approach (proactive testing of the population) early in the epidemic, using drive-through set-ups and testing booths. Through early testing (including of people with mild and non-symptomatic cases), tracing contacts, and quarantining of positive patients, they were able to target their responses and avoid the massive social and economic disruption experienced in most of Europe and North America. On a smaller scale, early mass screening in the town of Vo also helped it defy the wider trends that devastated northern Italy.

## SPECIFICATIONS OF USEFUL TESTS EARLY IN AN EPIDEMIC

The results of diagnostic tests must be sufficiently quick to inform quarantine decisions, but we often have to choose between accuracy and speed. False negatives are a particular concern as it implies sending an infected person out into the community where they can transmit the virus. Molecular methods are especially useful in the beginning of outbreaks because they can detect the pathogen even at the asymptomatic phase or with mild symptoms, at a time when patients may unknowingly be transmitting the virus. (Viral shedding in the case of COVID-19 has been found to be highest in the pre-symptomatic phase.) Antigen tests could also be useful early on but currently none are recommended as these tests generally require high levels of antigen expression [7]. In the case of Covid-19, there is the concern that patients with low infectious burden will be missed [8]. Antibody tests are useful later on for identifying who might have developed immunity to the virus and, as such, for possibly determining who could be allowed back to work. Despite doubts over sensitivity and specificity of the handheld immunity tests in late April 2020 [9], as of late June many companies are testing their employees for antibodies in order to optimally organize themselves as they resume normal activities.

**Figure:**
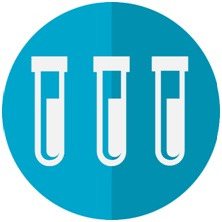
Photo: Biosamples (from Pixabay.com).

Timing of the launch of mass molecular testing is important. Once the disease has spread beyond the early stages, the majority of the population becomes part of the at-risk group. At that point, it becomes necessary to screen a very large number of individuals, which is both difficult and expensive. However, even these costs are small compared to having to close down economies. Even in countries that miss the chance for screening at the earliest stage of the outbreak, mass screening with molecular tests can still be used alongside social containment measures. Indeed “lockdown” measures can likely facilitate such testing and the results can be used to better prevent movement of infected individuals in and out of contained areas – thereby decreasing the porosity of the system. Crucially, at-home screening visits may also help define the necessary duration of the lockdown period (eg, 2-3 weeks) rather than letting it go for an undefined period of time as most western countries have had to do.

However, to react sufficiently quickly there needs to be a laboratory network in place that is capable of quickly developing new diagnostic tests based on the latest findings from counterparts, with high quality peer review. This could be built on existing projects such as EVD Laboratory-Net or EMERGE. To be as efficient as possible we may need mechanisms to activate collaboration amongst public and private research laboratories, especially where the two do not routinely interact. There also needs to be some maintenance of a hospital and clinic network ready and able to perform testing in close communication with the test developing group (potentially with some mediation by health authorities). In addition, national emergency regulatory measures need to allow flexibility in testing eligibility in response to transmission detected anywhere in the world, not just the country in question. Finally, the reporting of selected testing protocols to authorities along with initial cohort results should be considered. While most decision-making may need to be centralized, testing eligibility should be decentralized to the extent possible upon invoking of emergency measures.

## TIMING OF INDUSTRY PRODUCTION OF TESTS

Commercial production of pathogen-specific tests can rapidly increase the scale of testing. Commercial tests are often point-of-care, rather than laboratory-based tests. In the case of molecular diagnostics, commercial tests are thus far still laboratory-based but have advantages such as a lower level of required expertise and laboratory workup. (These latter characteristics will be increasingly important as COVID-19 spreads further through the developing world. At the time of writing, the number of COVID-19 cases in sub-Saharan Africa is low. Yet it is unclear why this is the case – whether it is due to the relatively young average age of the populations, climate-related factors, genetic factors or other reasons. It is also unclear whether propagation is simply delayed compared to model predictions.) Generally, companies are very experienced in producing tests for mass sale and, the larger companies in particular, have vast networks for broad distribution. The quick role out in testing in Germany is thought to be a result of market forces allowing these companies to do what they do best – in contrast to a slower, more restrained response in the UK [10].

But commercial tests are usually produced when there is a clear market for a product – ie, when there is sufficient demand stemming from prolonged presence of a disease or spreading of an epidemic – not at the start of the epidemic.

One catalyst for mass commercial production at the start of an epidemic is fast-moving (quick-to-commit) political leadership requesting help from industry. This can be expensive if there are few molecular diagnostics companies in the geographic area in which the government chooses to operate (eg, if there are pressures for domestic consumption), as the companies will have leverage in price negotiations. And of course it is risky in that – at that early point – it may still be unclear how useful the diagnostic will prove to be given the uncertainties regarding the epidemiology of the disease.

The other catalyst of mass production is the forward-looking, opportunity-grabbing entrepreneur. However, entrepreneurial activity independent of public support, is usually triggered only as data starts to come in, when uptake by health authorities seems likely and thereby lowers the risks of investment loss.

As the infection propagates and large-scale demand becomes clear, there are of course other market entrants. This may include producers encouraged to help facilitate early testing in parts of the world that the disease has yet to reach (eg, many of those working through the Foundation for Innovative New Diagnostics) or it can be merely production of specified tests for the sake of the portfolio, to keep up with competitors. Tests are also sometimes machine-specific or used as part of a reverse “razor and blade” business model, intended to boost sales of the major units. For example, Bosch recently announced that it has a rapid test that can provide results in 2.5 hours but this test can only be read by its recently approved Vivalytic machine [11]. Such sales models clearly don’t facilitate early mass screening either.

## DIFFERENCES IN TESTING CAPACITY

Singapore and South Korea in particular, foresaw the spread of the epidemic and began mass production of testing kits early on, with governments working closely with industry. In South Korea this was helped by commercial production, namely by Seegene, a small company of only 125 employees, specializing in molecular diagnostics. Development of the test started before the first case was even diagnosed in South Korea, and it was developed with the help of computer modelling in just a few weeks, using the published genetic sequence, without using a single sample of the virus itself [12].

Test kits were also developed early on in Germany where, even before the gene sequence for COVID-19 was published by the Chinese, a Berlin-based company TIB Molbiol designed a test kit based on other coronaviruses, published its protocol on the website of the WHO, and made the initial steps towards mass production of a usable test. Germany as a whole has been ahead of the rest of Europe, doing early screening and far more testing than its neighbours. As of mid-spring, the number of tests being conducted in Germany was said to be more than 500 000 per week, and as of the early days of summer, deaths there were several times lower than those in other nearby European countries of a similar population size [13].

## BARRIERS TO INDUSTRY RESPONSE

Industry response is of course fruitless where there is too much red tape prohibiting rapid market entry. In the US, traditional ‘home of the entrepreneur’, the ability of industry to respond to the COVID-19 epidemic was slowed by centralization and approval requirements. Throughout the early phase of the epidemic, hospitals were forced to send their samples to the US Centers for Disease Control itself for testing. The sending of faulty reagents by the International Reagent Resource (IRR) domestically further slowed the response. In theory any certified hospital or commercial laboratory could have used one of the protocols within the WHO repository published online and ordered primers from any company doing DNA synthesis. But, in this case, diagnostic tests had to wait for a second batch of reagents to be sent out by the IRR. At the time of writing the Food and Drug Administration had approved two commercial tests that should help scale up diagnostic capacity, however, we are now well beyond the point where screening can be used to narrow the epidemic response to infected individuals and thereby limit the economic impact. These commercial tests may, however, prove useful in the case of a second wave.

On the other end of the diagnostic readiness spectrum, Singapore began developing a test at the onset of the Chinese outbreak and hospitals were granted permission to use other approved PCR tests as long as they validated their first set of results with a national laboratory. Aside from regulation, the scale and timing of industry response can also be hindered by export restrictions. These tend to occur where national governments have failed to take early action and therefore become reluctant to allow potentially useful products out of their grasp. It should be noted that while such restrictions can badly damage international sales and future export negotiations of the companies in question, they do not necessarily imply domestic purchase. A company can very well find itself with unsold stock sitting in warehouses despite its usefulness.

Conversely, signalling early, adept epidemic response – including to the development of tests – by government can both directly and indirectly support domestic companies through internal consumption and export respectively. A clear example of this comes from the sales of diagnostics by a South Korean company to the state of Maryland in the US.

The (negative) public good nature of pandemics and the unreliability of national governments in the face of global competition for key supplies, suggest that we need more independent leadership in this area with financial sustainability that has no political strings attached. The WHO could in theory take a more important role in preparing for and responding to global pandemics–and indeed it has improved this function since the somewhat botched response to the Ebola epidemic of 2014. However, its financing structure makes it dependent on individual donor countries and lacking in the necessary independence that would allow it to act effectively in this situation. Only a truly independent organization could approach pandemic preparation in a sufficiently strategic way to have any chance of superseding the tendency of national governments to enforce export bans. It could for example help ensure regional surge capacity amongst transnational suppliers and support supply source diversification more generally. While the WHO could take a centralizing role in such activities, support would be needed from the other big agencies traditionally involved in procurement (eg, UNICEF, PAHO, Global Fund). (In the case of early COVID-19 diagnosis, continuity of supply of primers, probes and positive controls was reported as the biggest obstacle for test implementation [14].)

Independence from national politics is also necessary to address the needs of low-income countries during a pandemic – clearly an ethical imperative and a critical component of global infection control. To be effective, however, WHO would need more “teeth” to have the necessary influence [15]. Several experts suggest making international trade and financing agreements conditional on states’ cooperative behaviour in health matters and the fulfilling of commitments.

## LACK OF INSTITUTIONAL MEMORY

The ability to acquire tests quickly on a very wide scale is a matter of forethought and timely preparation – but it *is* possible. Seeing an epidemic unfold in a populated part of the planet with innumerable global trade and travel links would suggest that most pandemic preparation could be put at the top of any agenda in the developed world and quick action taken. Indeed pandemic response teams, where they are in place, would normally be in charge of setting in motion the necessary politico-industrial levers to bring to market what is needed, including tests. Yet new governing administrations often lack an understanding of history and over-estimate the ability of modern medicine to tackle novel threats, in part due to a lack of medical expertise at the higher echelons. Also – depending on the political structures (eg, make-up of civil servants vs political appointees), time since last affected by an outbreak locally, and ideology – institutional continuity is minimal, and lessons learnt can be lost between one outbreak and the next. This lack of memory is most tangible where it extends to the funding of critical research and development. For example, the serology and vaccine work done on SARS-CoV-1 would have probably been helpful to fighting SARS-CoV-2–indeed the serological tests designed for the former can pick up the latter–but much of the momentum was lost as funding dried up as soon as the first virus receded [16]. Clearly we need to better preserve clinical, epidemiological, and logistic experience from past epidemics. This requires continued support for multidisciplinary outbreak research, possibly through the creation of a discipline across the traditional sciences.

## CONCLUSIONS

Early broad screening allows for interventions to adapt to the microbiological reality, with tight quarantine of those who test positive but without necessarily shutting down entire economies. Limiting the impact of infectious disease epidemics is only possible if diagnostics are widely available and if health authorities are proactive in assessing the population-wide epidemiology of the disease early on. This requires flexibility in the regulations governing microbiology laboratories and is vastly aided by quick industry response. This is not necessarily difficult, but it does require political will, backed by institutional memory. Hopefully the message from the current economic difficulties will be clear: the costs of widespread screening in the earliest phase of an epidemic are minuscule compared to having to effectively shut down whole economies to contain it when it has spread across the population.

Finally, in all pandemics, national governments are forced to prioritize the needs of their national populations, and they will be less generous with other countries if they lack good demand estimates to determine domestic need–the data that only screening brings. Private companies are also less likely to produce other critical supplies if they don’t have a good picture of the potential demand that is gathered through screening. In other words, a coherent response to any infectious disease threat depends on early mass production and availability of tests globally. We need the organization and independent leadership to make this possible.
